# The Critical Role of Ferroptosis in Hepatocellular Carcinoma

**DOI:** 10.3389/fcell.2022.882571

**Published:** 2022-06-21

**Authors:** Fan Pan, Xinrong Lin, Liping Hao, Ting Wang, Haizhu Song, Rui Wang

**Affiliations:** Department of Medical Oncology, Affiliated Jinling Hospital, Medical School of Nanjing University, Nanjing, China

**Keywords:** ferroptosis, lipid peroxidation, GPx4, system xc-, iron metabolism, hepatocellular carcinoma

## Abstract

Liver cancer is the sixth most frequently diagnosed cancer and the third dominant cause of cancer death worldwide. Ferroptosis is characterized as an iron-dependent form of regulated cell death, with accumulation of lipid peroxides to lethal amounts. Evidences have showed that ferroptosis is closely associated with HCC, but the mechanisms are still poorly understood. In this review, we mainly summarize the roles of several typical molecules as well as radiotherapy in regulating the ferroptosis process in HCC. Chances are that this review may help address specific issues in the treatment of HCC.

## Introduction

Liver cancer is the sixth most frequently diagnosed cancer and the third dominant cause of cancer death worldwide. Globally about 905677 new liver cancer cases (4.7%) and 830180 new deaths (8.3%) were estimated to occur in 2020. Rates of both incidence and mortality of liver cancer show marked gender differences, with higher rates among men than among women (Sung et al., 2021). Hepatocellular carcinoma (HCC) comprises approximately 75–85% of liver cancer cases. HCC is closely associated with inflammation as most of HCC cases arise on the basis of hepatic injury and inflammation (Bishayee, 2014). HCC patients have previously suffered from chronic liver disease and cirrhosis, which are closely associated with major risk factors including HBV and/or HCV infection, excessive alcohol intake, non-alcoholic fatty liver disease (NAFLD), aflatoxin B1 exposure, diabetes, and obesity (El-Serag and Rudolph, 2007; Singal and El-Serag, 2015; Marengo et al., 2016; Wang et al., 2016). Increasing efforts have been devoted to addressing the problems associated with treatment of HCC such as drug resistance, but few progresses have been made.

Ferroptosis is characterized as an iron-dependent form of regulated cell death, with accumulation of lipid peroxides to lethal amounts. Ferroptosis was first noticed in 2003 when erastin induced death of engineered cell lines that expressed oncogenic RAS. The nuclear morphology of erastin-treated tumorigenic cells was distinct from that of apoptotic cells (Dolma et al., 2003). The mitochondrial morphologies of erastin-treated cells, such as loss of structural integrity, were different from cells of apoptosis, necrosis, and autophagy. Two isoforms of human mitochondrial voltage-dependent anion channels (VDAC2 and VDAC3) were confirmed to be associated with action of erastin. Besides, activation of RAS-RAF-MEK pathway was proved necessary for erastin function (Yagoda et al., 2007). By synthetic lethal screening, two other compounds, RAS-selective lethal 3 (RSL3) and RAS-selective lethal 5 (RSL5), were identified to display synthetic lethality to mutant RAS. Cell deaths induced by RSL3/5 were considered to be non-apoptotic and dependent on the RAS-RAF-MEK pathway. Similarities of erastin and RSL3/5-induced cell deaths were further found, as they were all inhibited by iron chelation and accompanied by accumulation of reactive oxygen species (ROS) (Yagoda et al., 2007; Yang and Stockwell, 2008). Finally, this oxidative, iron-dependent cell death was described as ferroptosis (Dixon et al., 2012). In this review, we are going to focus on ferroptosis, and summarize diverse regulatory mechanisms associated with ferroptosis in HCC. We hope that this review will be beneficial to solve problems regarding treatment of HCC.

## Biochemical Characteristics of Ferroptosis

**FIGURE 1:**
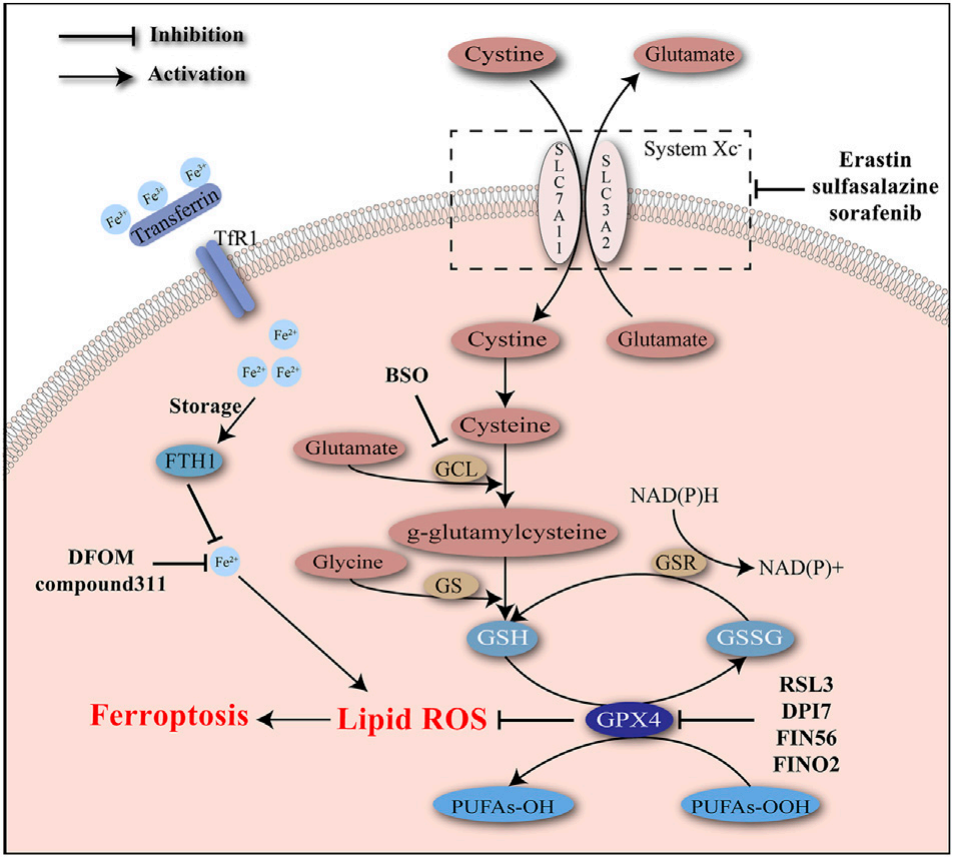
Iron accumulation and lipid peroxidation are two critical characteristics of ferroptosis and induce oxidative damage. Ferroptosis inducers act to intervene the key molecules involved in the regulatory network of ferroptosis.

Depletion of GSH, inactivation of GPX4, and accumulation of cellular iron and lipid ROS are identified as typical molecular events of ferroptosis (Figure 1).

### Depletion of GSH

Glutathione, as a significant scavenger of reactive species, protects cells from the stress of peroxides and other toxic compounds. Glutathione usually presents in the forms of reduced glutathione (GSH) and oxidized glutathione (GSSG). Conversion of GSH to GSSG mediated by glutathione peroxidases (GPXs) enables the reduction of peroxides (e.g., R–OOH) to their corresponding alcohols (e.g., R–OH). GSSG is later reduced back to GSH by GSSG reductase, accompanied with the consumption of NADPH. GPX4 is a unique subtype of GPXs, acting as a phospholipid hydro-peroxidase to reduce lipid peroxides to lipid alcohols. The endogenous redox cycle maintained by GSH and GPX4 is essential to defend lipid peroxidation (Lu, 2009; Brigelius-Flohé and Maiorino, 2013; Lu, 2013; Forcina and Dixon, 2019). Depletion of GSH and/or inhibition of GPX4 may lead to the breaking of redox balance and ultimately the accumulation of lipid peroxides.

During ferroptosis process induced by erastin, both GSH and GSSG were significantly depleted, accompanied with accumulation of lipid ROS. The increase in erastin concentration was correlated with the degree of GSH depletion. Lethal analogs of erastin were verified to deplete GSH more effectively than unlethal analogs of erastin. Besides, additional increasement of GSH was able to reverse cell death caused by erastin, indicating that GSH depletion was a pivotal factor in erastin-induced ferroptosis (Yang et al., 2014). GSH level was not influenced during oxidative cell death caused by some antioxidant inhibitors including DETC (an SOD inhibitor), DIA (a thiol-reactive reagent), and DCNB (a thioredoxin reductase inhibitor), suggesting that oxidative stress was not enough to induce ferroptosis.

A possible explanation for the GSH depletion during ferroptosis is the disturbance of GSH synthesis. Glutamate cysteine ligase (GCL) and GSH synthetase (GS) are rate-limiting enzymes of GSH synthesis that affect two separate ATP-dependent steps. GCL helps the combination of glutamate with cysteine into γ-glutamylcysteine, which later cooperates with glycine to synthesize GSH under the catalysis of GS (Forman et al., 2009; Lu, 2013). GCL is a heterodimer of catalytic (GCLC) and modifier (GCLM) subunits. Buthionine sulfoximine (BSO) is a robust inhibitor of GCL and causes extensive depletion of cellular GSH (Griffith and Meister, 1979; Meister, 1995). Previous studies have verified BSO’s cytotoxic effects on newborn rats and HCC cells (Mãrtensson and Meister, 1991; Lee et al., 2017). Another verified mechanism of suppressing GSH synthesis is the inhibition of the cystine/glutamate antiporter system Xc^–^. System Xc^–^is mainly composed of two subunits including SLC7A11 and SLC3A2, and widely existed in phospholipid bilayers. Cystine import and glutamate export are mediated by system Xc^–^at a ratio of 1:1 (Bannai and Kitamura, 1980). Cystine is then reduced to cysteine, providing raw materials for GSH synthesis. The actions of some molecules (e.g., erastin, sulfasalazine, sorafenib) to inhibit system Xc^–^have been confirmed. Excessive addition of glutamate also has a negative effect on system Xc^–^ (Forcina and Dixon, 2019).

### Inactivation of GXP4

Loss of GPX4 activity is viewed as another defining event of ferroptosis. As has been demonstrated, GPX4 is a unique subtype of GPXs and contributes to the elimination of lipid ROS (Forcina and Dixon, 2019). Knockdown of GPX4 was able to induce ferroptosis (Yang et al., 2014). Systemic depletion of GPX4 was noticed to be lethal to mice (Imai et al., 2003). RSL3 has been confirmed by affinity purification experiment to target GPX4 to induce ferroptosis (Yang et al., 2014). Mechanically, RSL3 covalently binds to the selenocysteine at the active site of GPX4 and inhibits its enzymatic activity irreversibly. A few DPI complexes (e.g., DPI7, DPI12, and DPI17) share the same binding site with RSL3 and act as inducers of ferroptosis (Yang et al., 2016).

Polyunsaturated fatty acyl moieties (PUFAs) are a class of lipid that are highly susceptible to oxidative damage. The bis-allylic protons of PUFAs are easily abstracted by hydrogen atoms, with the production of alkyl radicals which interact with molecular oxygen to generate peroxyl radicals. Peroxyl radicals could further interact with other PUFAs, leading to a chain reaction of lipid peroxidation. Analysis by LC tandem mass spectrometry (LC-MS/MS) showed that PUFAs were the potential target of GPX4 and functionally involved in ferroptosis. In addition, classic PUFAs such as arachidonic acid and linoleic acid were confirmed to sensitize RSL3-induced ferroptosis (Yang et al., 2016).

### Increased Level of Iron

High body iron stores were previously identified as risk factors of various cancers including HCC (Knekt et al., 1994; Stevens et al., 1994; Tang et al., 2020). Increased levels of iron were identified indispensable for ferroptosis process. Fenton chemistry refers to the formation of hydroxide (OH^−^) and hydroxyl radicals (OH•) from the reaction between Fe^2+^ and hydrogen peroxide (H_2_O_2_) (Harris and DeNicola, 2020). The autoxidation with Fenton chemistry was thought as a spontaneous, non-catalytic process which contributed to the lipid peroxidation (Capelletti et al., 2020).

Ferroptosis induced by RSLs was suppressed by iron chelators such as DFOM and compound311 dependent on their abilities of depleting iron, highlighting the significance of iron in ferroptosis. The iron responsive elements (IREs)/iron regulatory proteins (IRPs) regulatory system could regulate the expression of genes associated with iron uptake (transferrin receptor 1 (TfR1) and divalent metal transporter 1 (DMT1)), storage (ferritin), and release (ferroportin), maintaining the intracellular iron homeostasis (Recalcati et al., 2010). Expression of TfR1 was increased, while expression of an iron storage protein complex which includes ferritin heavy chain 1 (FTH1) and ferritin light chain (FTL) was decreased during ferroptosis, indicating the increase in iron uptake and decrease in iron storage during this process (Dolma et al., 2003). Erastin-induced ferroptosis requires the existence of transferrin, which carries iron and interacts with TfR1 to import iron. Transferrin was able to induce cell death in a dose-dependent manner, which could be inhibited by knockdown of TfR1 (Gao et al., 2015).

Numerous efforts have been devoted to clarifying the regulation of iron during ferroptosis. Heme oxygenase-1 (HO-1) is involved in removal of toxic heme and production of biliverdin, iron ions, and carbon monoxide. It was reported that HO-1 was required for iron reutilization in mammals (Tenhunen et al., 1969; Poss and Tonegawa, 1997). Recently HO-1 has been demonstrated to play a significant role in lipid peroxidation during ferroptosis. Erastin-induced cell death was inhibited by ZnPP which acted as a HO-1 inhibitor, with decreased level of lipid peroxidation. Besides, the by-product of HO-1 could promote cell death induced by erastin. HO-1 may contribute to the lipid peroxidation and the ferroptosis process, probably due to its regulatory role in iron metabolism and the Fenton chemistry (Kwon et al., 2015).

## Specific Inducers of Ferroptosis

Numerous studies have confirmed the effects of a series of substances in inducing ferroptosis. Generally, those substances can be divided into four groups according to their regulating properties. The first group includes erastin, sulfasalazine, and sorafenib, which inhibit the cystine/glutamate antiporter system Xc^–^ (Dixon et al., 2014). The second group of ferroptosis inducers, such as RSL3 and some DPI complexes (e.g., DPI7), could directly inhibit the active site of GPX4. The third group includes FIN56 that applies two approaches to induce ferroptosis. First, FIN56 promotes degradation of GPX4. Second, FIN56 binds to and activates the enzyme squalene synthase, giving rise to the depletion of coenzyme Q10 which serves as an endogenous antioxidant (Shimada et al., 2016). The last group of ferroptosis inducers comprises the 1,2-dioxolane FINO2, which can both directly oxidize the labile iron and indirectly inhibit enzymatic function of GPX4 (Gaschler et al., 2018).

Above, we have discussed the main molecular events as well as typical inducers of ferroptosis. To further elucidate the role of ferroptosis in HCC, we mainly introduce several typical molecules and the high-LET CI, and narrate the mechanisms of how they are involved in regulation of ferroptosis in HCC.

### The Nuclear Factor Erythroid 2-Related Factor 2 (Nrf2)

Nrf2 is a critical regulator of antioxidant response. Mechanically, Nrf2 acts by heterodimerizing with small Maf proteins and binding to antioxidant responsive elements (ARE) to regulate genes involved in antioxidant homeostasis (Itoh et al., 1997; Ma, 2013). Kelch-like ECH-associated protein 1 (Keap1) is a negative regulator of Nrf2. Under unstressed conditions, Keap1 acts as an adaptor of the ubiquitin ligase complex and mediates the degradation of Nrf2 through ubiquitin-proteasome pathway (Itoh et al., 1999; Cullinan et al., 2004; Kobayashi et al., 2004). Oxidative stress could inactivate Keap1 and promote the stabilization of Nrf2 (Wakabayashi et al., 2004; Kobayashi et al., 2006). A selective substrate for autophagy, p62 (also called sequestosome 1), was reported to competitively interact with the Nrf2-binding site of Keap1, resulting in the indirect activation of Nrf2 (Komatsu et al., 2010). Recently, this p62-Keap1-Nrf2 pathway was believed to be involved in the regulation of ferroptosis induced by erastin and sorafenib in HCC cells (Sun et al., 2016b).

Erastin, sorafenib, and BSO were able to increase the expression of Nrf2 in HCC cells, with decreased expression of Keap1 and increased interaction between p62 and Keap1. The upregulated Nrf2 was thought to contribute to the resistance to ferroptosis. Knockdown of Nrf2 enhanced the decrease in cell viability induced by erastin and sorafenib in HCC cells, with increased defining events of ferroptosis (e.g., GSH depletion, lipid ROS accumulation, and increased level of iron), which could be reversed by inhibitors of ferroptosis other than inhibitors of apoptosis and necroptosis. Knockdown of Keap1 had the opposite influence. Several genes were identified as target genes of Nrf2 following treatment of erastin and sorafenib, including quinone oxidoreductase 1 (NQO1), heme oxygenase-1 (HO1), and ferritin heavy chain 1 (FTH1), which were involved in ROS and iron metabolism. *In vivo* experiments further indicated that the ferroptosis process could be enhanced by Nrf2 silencing, with the HCC tumor eliminated more efficiently (Sun et al., 2016b).

In conclusion, the p62-Keap1-Nrf2 antioxidative signaling pathway is involved in ferroptosis induced by erastin and sorafenib in HCC cells, probably through regulating genes associated with ROS and iron metabolism. Decreased expression of Nrf2 could increase the susceptibility of HCC cells to ferroptosis induced by erastin and sorafenib. In addition, Nrf2 has previously been associated with induction of SLC7A11 gene by electrophilic agents, indicating its role in GSH metabolism and another method of regulating ferroptosis process (Sasaki et al., 2002).

### Sigma-1 Receptor (S1R)

Sigma-1 Receptor (S1R) is a non-opioid receptor which exhibits molecular chaperone activity and is widely expressed in many organs including liver (Lievens and Maurice, 2021). S1R is involved in antioxidant homeostasis through activation of ARE which could be regulated by Nrf2. Knockdown of S1R has been associated with decreased function of system Xc^–^and reduced expression of SLC7A11, with increased endogenous ROS levels (Nguyen et al., 2003; Pal et al., 2012; Wang et al., 2015). SLC7A11 was identified by integrated analysis as one of the potential targets for the prognosis and diagnosis of HCC. Both mRNA level and expression of SLC7A11 were upregulated in HCC tumors. Inhibition of SLC7A11 rendered lipid peroxidation in HCC cells, with increased levels of ROS (Tang et al., 2020).

It was found that sorafenib increased the expression of S1R and induced most of S1Rs away from nucleus in HCC cells, which could be suppressed by ferrostatin-1, suggesting the potential regulatory role of S1R in ferroptosis. Suppression of S1R significantly enhanced cell death of HCC cells induced by both erastin and sorafenib, which could be inhibited by ferroptosis inhibitors (e.g., ferrostatin-1) other than inhibitors of apoptosis or necroptosis. Knockdown of S1R could sensitize HCC tumors to sorafenib *in vivo*. In S1R knockdown HCC cells, increased levels of MDA (a typical product of lipid peroxidation) and iron were noticed following the treatment of erastin and sorafenib, suggesting that S1R probably modulates lipid peroxidation and iron metabolism to affect ferroptosis. Iron metabolism associated genes (e.g., FTH1) and lipid peroxidation associated genes (e.g., HO-1 and GPX4) were inhibited in S1R knockdown HCC cells following erastin and sorafenib treatment (Bai et al., 2019).

Briefly, S1R is thought as a negative regulator of ferroptosis in human HCC cells through involving in ROS and iron metabolism. Inhibition of S1R is able to sensitize HCC cells to erastin and sorafenib both *in vitro* and *in vivo*.

### Heat Shock Protein Beta-1 (HSPB1)

The heat shock proteins (HSPs) are thought to function as molecular chaperones and be involved in various biological processes, including the folding, assembly, transportation, and degradation of proteins. Typically, HSPs are essential to dealing with the abnormally folded proteins within the cells (Georgopoulos and Welch, 1993). It has been demonstrated that HSPs are involved in the proliferation, migration, invasion, and metastasis of cancer cells, as well as resistance to various anti-cancer drugs (Okuno et al., 2016; Wu et al., 2017). Heat Shock Protein Beta-1 (HSPB1) (also known as HSP27), as a member of small HSPs, is an ATP-independent molecular chaperone and exhibits strong cytoprotective properties (Garrido et al., 2006).

HSPB1 is viewed as a prognostic biomarker of HCC. According to statistics from The Cancer Genome Atlas (TCGA) database, expression of HSPB1 was significantly increased in HCC tissues. HCC patients with high HSPB1 levels showed worse overall survival rates and poor prognosis. Overexpression of HSPB1 was closely correlated with migration and invasion of HCC cells, as well as the *in vivo* metastasis (Zhang et al., 2016; Long et al., 2021). Besides, HSPB1 was identified as a hub gene in the regulatory network associated with HCC progression and ferroptosis (Fei et al., 2021).

HSPB1 has been reported to be a negative regulator of ferroptosis (Sun et al., 2015). Inhibition of HSPB1 and HSF1 which acted as an upstream regulator of HSPB1 enhanced erastin-induced ferroptosis, accompanied with increased levels of cellular iron and lipid ROS. The process could be rescued by deferoxamine (an iron chelator) and ferrostain-1 (a ferroptosis inhibitor). The phosphorylation of HSPB1 could be mediated by protein kinase C (PKC) and set a protective effect on erastin-treated cancer cells, probably due to its involvement in actin polymerization and reorganization which inhibits iron uptake. Knockdown of HSPB1 was associated with increased expression of TfR1 and mildly decreased expression of FTH1 (Lavoie et al., 1993; Sun et al., 2015).

In summary, HSPB1 is clinically correlated with poor prognosis in HCC patients. Overexpression of HSPB1 in HCC cells is closely linked to migration and invasion *in vitro* and metastasis *in vivo*. HSPB1 has been identified by bioinformatic strategies as a hub gene in the regulatory network associated with HCC progression and ferroptosis. Mechanically it may negatively regulate ferroptosis process through involvement in iron metabolism.

### Metallothionein-1G (MT-1G)

Metallothioneins (MTs) are a superfamily of intracellular, low molecular weight, cysteine-rich polypeptides. The unique structures of MTs enable their metal binding function and redox capabilities. MTs have been considered important for adapting to cellular stress from oxyradicals, toxic metals, inflammation, infection and low Zn nutrition (Sun et al., 2016a). MTs are closely associated with carcinogenesis, progression, and drug resistance of cancers including HCC (Deng et al., 1998; Gumulec et al., 2014; Si and Lang, 2018). The MT-1, which is among the most widely expressed isoforms in the body, includes 13 subtypes in which Metallothionein-1G (MT-1G) has been associated with sorafenib resistance in HCC through inhibiting ferroptosis (Sun et al., 2016a).

Both mRNA level and protein expression of MT-1G were increased in HCC cells by treatment of sorafenib. Nrf2 was involved in this process that silencing of Nrf2 was able to inhibit MT-1G mRNA induced by sorafenib. Cell viabilities were decreased significantly by inhibition of MT-1G and Nrf2 following the treatment of sorafenib, which could be rescued by ferroptosis inhibitors such as ferrostatin-1 and liprostatin-1. Knockdown of MT-1G was also noticed to enhance cell death induced by other inducers of ferroptosis such as erastin, indicating the involvement of MT-1G in regulation of ferroptosis. In addition, following treatment of erastin and sorafenib, knockdown of MT-1G was able to deplete GSH and increase the level of MDA which is an end product of lipid peroxidation. *In vivo* experiments further demonstrated that inhibition of MT-1G significantly improved efficacy of sorafenib in treatment of HCC, with decreased GSH levels and increased expression of PTGS2 which served as a marker to assess ferroptosis (Sun et al., 2016a).

In summary, increased expression of MT-1G is induced by sorafenib via a Nrf2-dependent manner in HCC cells. MT-1G is involved in resistance to sorafenib in HCC, probably through inhibiting GSH depletion and suppressing the ferroptosis process.

### P53

*TP53* is a well-known tumor suppressor gene that is closely associated with cell cycle arrest, cellular apoptosis, and cellular senescence. Inactivation of *TP53* was observed in more than half of sporadic human cancers including HCC (Junttila and Evan, 2009; Bieging et al., 2014; Calderaro et al., 2017).

In human HCC cells, a nonsynonymous single-nucleotide polymorphism at codon 47 of *TP53* (also known as S47) was reported to disrupt the transactivate functions of p53 and induce resistance to ferroptosis (Jennis et al., 2016), suggesting p53’s role in inducing ferroptosis. *TP53* was involved in the transcription of *SLC7A11* which is a critical component of system Xc^–^ (Koppula et al., 2021). Chromatin immunoprecipitation revealed that p53 protein could be recruited to the promoter region of the *SLC7A11* gene. Activation of p53 significantly reduced the mRNA level and the expression of SLC7A11, leading to the inactivation of system Xc^–^and reduced cellular level of GSH in cancer cells. Overexpression of p53 resulted in increased cellular sensitivity to erastin, which could be inhibited by ferrostatin-1 and reversed by upregulation of SLC7A11, suggesting that p53 acted to sensitize cells to ferroptosis via SLC7A11 (Jiang et al., 2015). Indeed, in HCC cells, inhibition of *TP53* was reported to increase expression of SLC7A11 following treatment of sorafenib (Zheng et al., 2022).

In conclusion, disfunction of p53 is correlated with resistance to ferroptosis in human HCC, probably through transcriptionally regulating the expression of SLC7A11.

### Yes-Associated Protein (YAP)/ Transcriptional Co-Activator With PDZ-Binding Motif (TAZ)

Yes-Associated Protein (YAP)/TAZ are effectors of Hippo signaling pathway and transcriptionally regulate the initiation, progression, and metastasis of cancer (Piccolo et al., 2014; Zanconato et al., 2016; Sun and Chi, 2021). TAZ was identified by a genome-wide shRNA-mediated synthetic lethality screen as an intrinsic driver of sorafenib resistance in HCC cells. High levels of YAP and TAZ were found in sorafenib-resistant HCC cells. Suppression of YAP/TAZ rendered lipid peroxidation and enhanced cell death following the treatment of sorafenib and erastin, which could be prevented by ferrostatin-1. Moreover, forced expression of YAP was able to prevent cell death induced by sorafenib, suggesting that YAP/TAZ act as inhibitors of ferroptosis in HCC cells (Gao et al., 2021).

It was postulated that YAP/TAZ target the SLC7A11 to affect ferroptosis in HCC cells, thus contributing to resistance to sorafenib (Gao et al., 2021). Expression of SLC7A11 was positively correlated with expression of YAP in HCC patient samples. Knockdown of YAP and TAZ were confirmed to reduce the mRNA levels and the expression of SLC7A11 in HCC cells, with enhanced cell death induced by sorafenib. Forced expression of SLC7A11 was able to set a protective effect on YAP/TAZ-deficient HCC cells, suggesting that YAP/TAZ’s influences on ferroptosis in HCC cells was through regulation of SLC7A11. Moreover, a TEAD binding motif on the promoter sequence of SLC7A11 was identified among the binding site of YAP/TAZ.

Activating transcription factor 4 (ATF4) is a critical mediator of oxidative homeostasis and has been validated to inhibit ferroptosis induced by sorafenib, erastin, and RSL3 via acting on SLC7A11 (Chen et al., 2017). ATF4 was found to be a driver of SLC7A11 expression in sorafenib-resistant HCC cells. Evidences showed that ATF4 inhibition promoted the ferroptosis process induced by sorafenib in HCC with increased lipid peroxidation, decreased GSH, and decreased cell viability, which could be suppressed by ferrostatin-1 as well as forced expression of SLC7A11, suggesting that ATF4 was involved in resistance to ferroptosis in HCC through the regulation of SLC7A11. Interestingly, ATF4 was associated with YAP/TAZ that activation of YAP/TAZ led to the nuclear translocation of ATF4 to transcriptionally increase its expression, as well as suppression of ATF4’s ubiquitination and proteasomal degradation, ultimately stabilizing ATF4 protein (Gao et al., 2021).

In short, YAP/TAZ are negative regulators of ferroptosis in HCC cells through activating SLC7A11 dependent on the TEAD motif, and through interacting with ATF4 which is a promotor of SLC7A11 expression.

### Transforming Growth Factor Beta-1 

Transforming Growth Factor Beta-1 (TGF-β1) has previously been associated with GSH metabolism TGF-β1-induced apoptosis in hepatocyte was accompanied with inactivation of GCLC which is catalytic subunit of a rate-limiting enzyme of GSH synthesis (Franklin et al., 2003). Those results gave rise to the hypothesis that TGF-β1 could contribute to the ROS metabolism and the ferroptosis process.

HCC cell lines with an early TGF-β1 gene signature include PLC/PRF/5, Huh7, Huh6, and HepG2, which exhibit cytostasis and apoptosis in response to TGF-β1 (Dzieran et al., 2013). Expression of SLC7A11 was downregulated in those cells following the treatment of TGF-β1, which could be inhibited by knockdown of Smad3. Forced expression of Smad3, similar to TGF-β1 treatment, led to the repression of SLC7A11 expression in HCC cells, and additional treatment of TGF-β1 was able to enhance the repression, indicating that TGF-β1 suppressed expression of SLC7A11 via Smad3. TGF-β1-induced SLC7A11 inhibition was accompanied with increased levels of ROS which was alleviated by forced expression of SLC7A11. C11-BODIPY probe was used to measure degree of lipid peroxidation. It turned out that the increased lipid peroxidation induced by TGF-β1 could be inhibited by ferrostatin-1 (a ferroptosis inhibitor) and deferoxamine (DFOA, an iron chelator), but cell viability was not clearly affected which was probably due to the compensatory antioxidant pathways discussed below. Interestingly, TGF-β1 could enhance the reduced cell viability induced by RSL3, suggesting that TGF-β1 could sensitize GPX4 to RSL3 and thus promote the ferroptosis in HCC cells (Kim et al., 2020).

In short, TGF-β1 is able to inhibit expression of SLC7A11 dependent on Smad3 in HCC cells with an early TGF-β1 signature and promote lipid peroxidation in a non-lethal manner. In addition, TGF-β1 probably contributes to ferroptosis that it could enhance the reduced cell viabilities induced by RSL3 in HCC cells.

### Interferon Gamma (IFNγ)

IFNγ is well-known as a pleiotropic immunomodulator. The immune response induced by IFNγ involves T helper (Th) CD4^+^ T cells and CD8^+^ cytolytic T cells, and is paradoxically associated with cancers (Castro et al., 2018; Alspach et al., 2019). It was found that IFNγ was closely associated with ferroptosis. IFNγ could be derived from immunotherapy-activated CD8^+^ T cells and give rise to ferroptosis through negatively regulating SLC7A11 (Lang et al., 2019). Regulation by IFNγ on ferroptosis was also reported in HCC cells.

Pretreatment of IFNγ was noticed to enhance ferroptosis induced by erastin and RSL3 in HCC cells, with increased accumulation of ROS which could be reversed by ferrostatin-1 (Kong et al., 2021). Mitochondria was previously linked to ferroptosis that cysteine deprivation induced the hyperpolarized potential on mitochondrial membrane and subsequent accumulation of lipid peroxides (Gao et al., 2019). Actually, pretreatment of IFNγ was found to increase mitochondria oxidation in HCC cells following treatment of erastin. Besides, this combination of IFNγ with ferroptosis inducers (e.g., erastin and RSL3) led to increased cell cycle arrest accompanied by decreased activity of cyclinD1/CDKs complex, suppressing proliferation of HCC cells. System Xc^–^was thought as target of IFNγ to regulate ferroptosis since expressions of SLC7A11 and SLC3A2 were both significantly decreased. IFNγ mediated JAK/STAT1/IRF1 activation was considered critical to this process since STAT1 and STAT3 were verified to bind to promoter sites of SLC7A11 and transcriptionally reduce its expression (Motaghed et al., 2014). Correspondingly, increased levels of p-STAT1, p-STAT3, and IRF1 were observed in HCC cells following the synergic treatment of IFNγ and ferroptosis inducers (Kong et al., 2021).

In short, IFNγ could negatively regulate the expression of SLC7A11 and SLC3A2 through JAK/STAT pathway in HCC cells, and increase mitochondria oxidation, thus sensitizing HCC cells to ferroptosis.

### Retinoblastoma (Rb)

Retinoblastoma (Rb) is known as the first tumor suppressor gene (Dyson, 2016). Through inhibition of E2F, the Rb protein could arrest cells in G1 phase of cell cycle (Burkhart and Sage, 2008). Loss of Rb is involved in initiation of cancers including retinoblastoma, HPV, small-cell lung carcinoma, and HCC (Meuwissen et al., 2003; Doorbar, 2006; Mayhew et al., 2007; Munakata et al., 2007; Dimaras et al., 2008). In HCC, invalidation of Rb could be achieved from gene mutation, epigenetic modification, and molecular regulation, and give rise to the carcinogenesis process (Laurent-Puig and Zucman-Rossi, 2006; Mayhew et al., 2007; Anwar et al., 2014).

In addition to its role in initiating HCC, loss of Rb was believed to be related to sensitivity to sorafenib of HCC cells. Decreased viability of sorafenib-treated HCC cells was correlated with loss of Rb. Besides, in murine HCC xenografts, knockdown of Rb strongly enhanced sorafenib’s efficacy in eradicating HCC tumors. Inhibitors of ferroptosis (e.g., DFX and Fer-1) were able to rescue Rb-knockdown HCC cells from cytotoxic effects of sorafenib, suggesting the roles of ferroptosis in sensitizing Rb-knockdown cells to sorafenib treatment. Knockdown of Rb was also capable to enhance the cytotoxic effect on HCC cells by erastin which was another inducer of ferroptosis and structurally different from sorafenib, further indicating that loss of Rb may sensitize HCC cells to ferroptosis (Louandre et al., 2015; Liang et al., 2019). Levels of ROS, especially those from mitochondrial production, were noticed increased by knockdown of Rb, but levels of GSH and GPX4 were not clearly affected. Cytotoxicity of sorafenib on HCC cells could be alleviated by inhibitors against mitochondrial respiratory chain. Considering sorafenib’s effect on morphological remodeling of mitochondria, loss of Rb probably acted to increase mitochondrial ROS to promote the cytotoxic effect of sorafenib on HCC cells (Louandre et al., 2015).

In conclusion, loss of Rb probably sensitizes ferroptosis induced by sorafenib in HCC cells through increasing the mitochondrial production of ROS.

### Radiotherapy

Radiotherapy has recently been associated with ferroptosis in HCC. The high linear energy transfer (LET) carbon ions (CI) were widely employed in the treatment of tumors. The sharp Bragg peak induced by high-LET CI could give rise to improved efficacy of tumor treatment without increasing the damage to normal tissues (Kamada et al., 2015; Nickoloff, 2015). The PKR-like ER kinase (PERK) is a transmembrane sensor that is involved in the regulation of unfolded protein response (UPR) to deal with ER stress induced by CI irradiation (Nagelkerke et al., 2013; Bhat et al., 2017). CI irradiation was reported able to increase expression of p53 via PERK (Zheng et al., 2022). P53 helped link CI irradiation to both apoptosis and ferroptosis in HCC cells. P53 could upregulate the expressions of PUMA and NOXA which were typical Bcl-2 family proteins that promote apoptosis (Edlich, 2018; Zheng et al., 2022). As has been discussed, disfunction of p53 contributed to resistance to ferroptosis in HCC cells (Jennis et al., 2016). Knockdown of *TP53* was able to increase expression of SLC7A11 in HCC cells and lead to reduced sensitivity to CI irradiation. CI irradiationpromoted lipid peroxidation and the shrunken mitochondria with increased membrane density and decreased crest following treatment of sorafenib in HCC cells, which were considered as morphological characteristics of ferroptosis. However, the reduced expression of SLC7A11 was found to be induced by combined treatment of CI irradiation and sorafenib other than CI irradiation alone, and could be rescued by ferrostatin-1 (Zheng et al., 2022).

In conclusion, high-LET CI is able to enhance the ferroptosis process following the treatment of sorafenib in HCC by negatively regulating expression of SLC7A11, probably through the upregulation of p53 via PERK.

## Discussion

**FIGURE 2:**
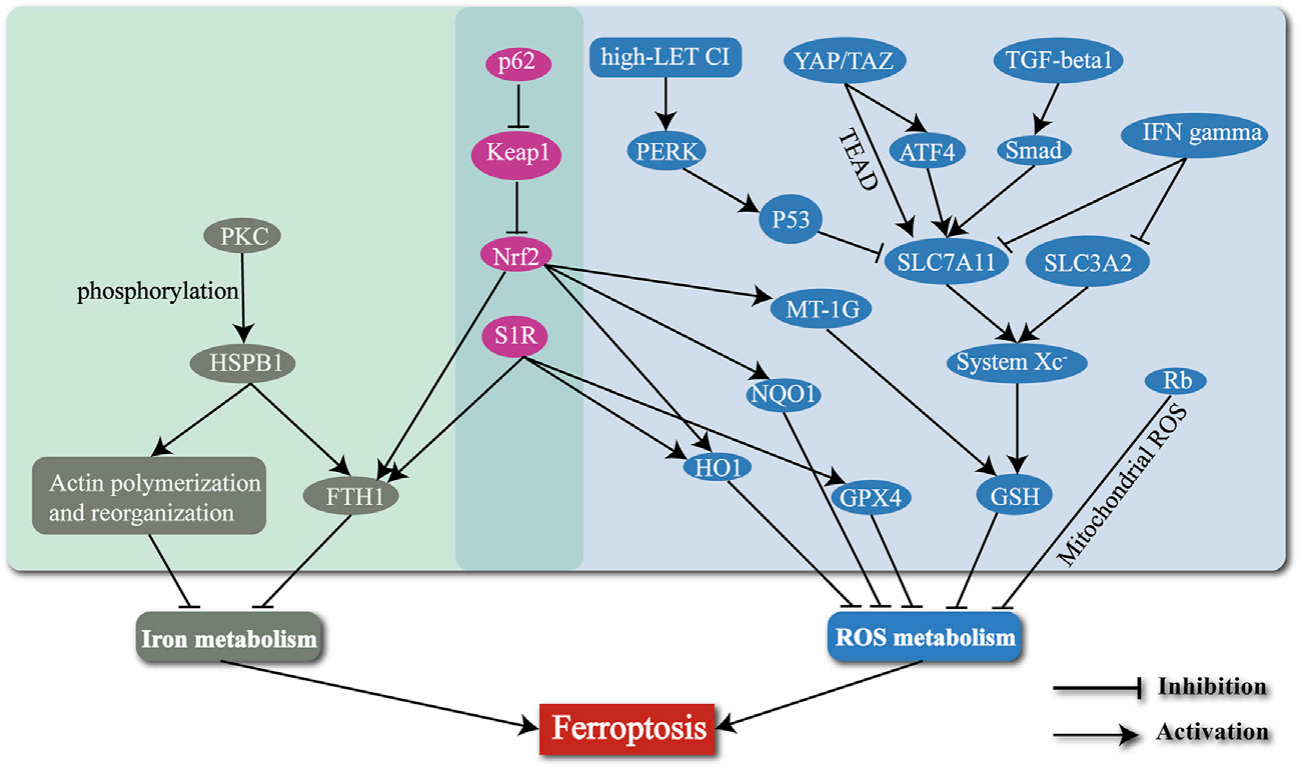
The mechanisms of regulating the ferroptosis process in HCC by several typical molecules and radiotherapy.

Ferroptosis is a newly defined form of regulated cell death, with iron-dependent accumulation of lipid peroxides to lethal amounts. Hepatic ferroptosis has been associated with liver diseases including nonalcoholic steatohepatitis (NASH) (Tsurusaki et al., 2019), APAP-induced acute liver failure (Yamada et al., 2020), and HCC. The high incidence and mortality of HCC have made it a serious hazard to human health worldwide (Sung et al., 2021). Increasing resistance to targeted therapies for HCC has become a challenge. Ferroptosis may provide new strategies to prevent the acquired resistance to those therapies. In this review, we mainly concentrate on several typical molecules including Nrf2 (Sun et al., 2016b), S1R (Bai et al., 2019), HSPB1 (Sun et al., 2015), MT-1G (Sun et al., 2016a), p53 (Jiang et al., 2015; Jennis et al., 2016), YAP/TAZ (Gao et al., 2021), TGF-β1 (Kim et al., 2020), IFNγ (Kong et al., 2021), Rb (Louandre et al., 2015), and the high-LET CI (Zheng et al., 2022) (Table 1), and narrate how they are involved in regulation of ferroptosis in HCC (Figure 2). Summary of these mechanisms is expected to help solve problems associated with clinical treatment of HCC, including resistance to sorafenib.

**TABLE 1:** Some typical molecules and radiotherapy are involved in regulation of ferroptosis in HCC.

	Downstream targets	Associated biological events	Influences	References
Molecules				
Nrf2	FTH1、HO1、NQO1	Iron and ROS metabolism	Resistant to ferroptosis and sorafenib	Sun et al. (2016b)
S1R	FTH1、HO1、GPX4	Iron and ROS metabolism	Resistant to ferroptosis and sorafenib	Bai et al. (2019)
HSPB1	FTH1、Actin polymerization and reorganization	Iron metabolism	Resistant to ferroptosis and sorafenib	Sun et al. (2015)
MT-1G	GSH	ROS metabolism	Resistant to ferroptosis	Sun et al. (2016a)
P53	SLC7A11	ROS metabolism	Resistant to ferroptosis	(Jiang et al., 2015; Jennis et al., 2016)
YAP/TAZ	SLC7A11	ROS metabolism	Sensitive to ferroptosis	Gao et al. (2021)
TGF-β1	SLC7A11	ROS metabolism	Sensitive to ferroptosis	Kim et al. (2020)
IFNγ	SLC7A11 SLC3A2	ROS metabolism	Sensitive to ferroptosis	Kong et al. (2021)
Rb	Mitochondrial ROS	ROS metabolism	Resistance to ferroptosis and sorafenib	Louandre et al. (2015)
Radiotherapy				
High-LET CI	PERK、P53	ROS metabolism	Sensitive to ferroptosis	Zheng et al. (2022)

Sorafenib is a multi-kinase inhibitor of HCC and the only drug approved by FDA for first-line treatment of advanced HCC (Siegel et al., 2010). Clinical trials have validated sorafenib’s efficacy in prolonging the overall survival (OS) of advanced HCC patients (Llovet et al., 2008). However, acquired resistance to sorafenib has become an obstacle of HCC treatment. Evidences have demonstrated that sorafenib served as one of the agents that could induce ferroptosis and thus contribute to treatment of tumors. Sorafenib-induced cell death could be rescued by ferroptosis inhibitors including ferrostatin-1, deferoxamine, and N-acetyl-l-cysteine (Louandre et al., 2013; Wang et al., 2021). Similar to erastin, sorafenib was thought to inhibit system Xc^–^and cause GSH depletion, finally giving rise to accumulation of lipid peroxides (Dixon et al., 2014). This review describes some regulatory pathways partly explaining how ferroptosis affect sorafenib-resistance in HCC. It is reasonable that intervening those pathways may help solve problems of sorafenib-resistance during HCC treatment.

Radiotherapy, which uses targeted delivery of ionizing radiation (IR), is a mainstay treatment for localized solid cancers (Delaney et al., 2005; Chen, 2019). Radiotherapy functions by eliciting various types of DNA damage (Baidoo et al., 2013), inducing radiolysis of cellular water, and stimulating oxidative enzymes (Azzam et al., 2012), subsequently leading to damage to nucleic acids, lipids, and proteins. The curative radiotherapy has clinically been applied for decades. The applications of novel accelerators for the delivery of proton and heavy-ion charged-particle therapy have accessed therapeutic benefits (Thariat et al., 2013). However, it should be mentioned that radiation exposure usually leads to acute tissue inflammation which can bring about further DNA DSBs and damages to cells and tissues (Westbrook et al., 2012). A lot of efforts are needed to explore the ratio between an optimal dose in the tumor and the lowest dose possible in the organs at risk (OAR). In this review, we talked about the regulation of ferroptosis by high-LET CI. Actually, radiotherapy may interact with immunotherapy to affect the ferroptosis (Lang et al., 2019). T cell responses, which were typical characteristics of immunotherapy, were thought as drivers to increase efficacy of radiotherapy via IFNγ (Lee et al., 2009). IFNγ alone was able to downregulate expressions of SLC7A11 and induce ferroptosis. Synergic treatments of IFNγ and radiotherapy could enhance this influence. Anti-CTLA4, as one of the immune checkpoint inhibitors, was able to improve the efficacy of radiotherapy, with enhanced tumoral lipid peroxidation and increased CD8^+^ T cells within tumors. The liproxstatin-1 could suppress those effects induced by anti-CTLA4, further indicating the significant roles of ferroptosis in synergic treatment of radiotherapy and immunotherapy (Lang et al., 2019).

Controversially, compensatory antioxidant pathways may complicate regulation of ferroptosis. As has been demonstrated, BSO is an inhibitor of GCL that suppresses the synthesis of GSH (Griffith and Meister, 1979; Meister, 1995). BSO has been demonstrated to induce ferroptosis in hepatic stellate cells (Zhang et al., 2018). GCLC and GCLM are two subunits of GCL. Previous studies showed that knockout of GCLC in mice led to complete GSH deficiency and the embryonic death (Dalton et al., 2000). However, cellular GSH was significantly reduced but persisted after knockout of GCLM (Harris et al., 2015). Interestingly, GCLM-knockout cells were noticed able to survive after further treatment of BSO. Functions of BSO in eliminating established tumors were limited. It was also proposed that most cancer cell lines are largely unaffected by GSH depletion (Harris et al., 2019). A possible explanation for this is the existence of alternative antioxidant pathways. The thioredoxin (TXN) antioxidant pathway is able to reduce ROS in a GSH-independent manner (Holmgren and Lu, 2010). Actually, mRNA of thioredoxin reductase 1 (TXNRD1) was upregulated in GCLM-knockout mice. Although treatment of BSO and auranofin (AUR, an inhibitor of TXNRD) alone had limited effect on survival of cancer cells, combined treatment of BSO and AUR led to enhanced cell death (Harris et al., 2015). Knockout of GSR, Trx1 or TrxR1 have also been verified unlethal to mouse liver (Rogers et al., 2004; Prigge et al., 2017). The compensatory antioxidant pathways may account for the limited effect of GSH depletion on cancer cells. More efforts are needed to explore the complicated role of GSH depletion during ferroptosis and its clinical applications.

## References

[B1] Alspach E. Lussier D. M. Schreiber R. D. (2019). Interferon γ and its Important Roles in Promoting and Inhibiting Spontaneous and Therapeutic Cancer Immunity. Cold Spring Harb. Perspect. Biol. 11 (3), a028480. 10.1101/cshperspect.a028480 29661791 PMC6396335

[B2] Anwar S. L. Krech T. Hasemeier B. Schipper E. Schweitzer N. Vogel A. (2014). Deregulation ofRB1expression by Loss of Imprinting in Human Hepatocellular Carcinoma. J. Pathol. 233 (4), 392–401. 10.1002/path.4376 24838394

[B3] Azzam E. I. Jay-Gerin J.-P. Pain D. (2012). Ionizing Radiation-Induced Metabolic Oxidative Stress and Prolonged Cell Injury. Cancer Lett. 327 (1-2), 48–60. 10.1016/j.canlet.2011.12.012 22182453 PMC3980444

[B4] Bai T. Lei P. Zhou H. Liang R. Zhu R. Wang W. (2019). Sigma‐1 Receptor Protects against Ferroptosis in Hepatocellular Carcinoma Cells. J. Cell Mol. Med. 23 (11), 7349–7359. 10.1111/jcmm.14594 31507082 PMC6815844

[B5] Baidoo K. E. Yong K. Brechbiel M. W. (2013). Molecular Pathways: Targeted α-Particle Radiation Therapy. Clin. Cancer Res. 19 (3), 530–537. 10.1158/1078-0432.ccr-12-0298 23230321 PMC3563752

[B6] Bannai S. Kitamura E. (1980). Transport Interaction of L-Cystine and L-Glutamate in Human Diploid Fibroblasts in Culture. J. Biol. Chem. 255 (6), 2372–2376. 10.1016/s0021-9258(19)85901-x 7358676

[B7] Bhat T. A. Chaudhary A. K. Kumar S. O’Malley J. Inigo J. R. Kumar R. (2017). Endoplasmic Reticulum-Mediated Unfolded Protein Response and Mitochondrial Apoptosis in Cancer. Biochimica Biophysica Acta (BBA) - Rev. Cancer 1867 (1), 58–66. 10.1016/j.bbcan.2016.12.002 PMC5272864 27988298

[B8] Bieging K. T. Mello S. S. Attardi L. D. (2014). Unravelling Mechanisms of P53-Mediated Tumour Suppression. Nat. Rev. Cancer 14 (5), 359–370. 10.1038/nrc3711 24739573 PMC4049238

[B9] Bishayee A. (2014). The Inflammation and Liver Cancer. Adv. Exp. Med. Biol. 816, 401–435. 10.1007/978-3-0348-0837-8_16 24818732

[B10] Brigelius-Flohé R. Maiorino M. (2013). Glutathione Peroxidases. Biochimica Biophysica Acta (BBA) - General Subj. 1830 (5), 3289–3303. 10.1016/j.bbagen.2012.11.020 23201771

[B11] Burkhart D. L. Sage J. (2008). Cellular Mechanisms of Tumour Suppression by the Retinoblastoma Gene. Nat. Rev. Cancer 8 (9), 671–682. 10.1038/nrc2399 18650841 PMC6996492

[B12] Calderaro J. Couchy G. Imbeaud S. Amaddeo G. Letouzé E. Blanc J.-F. (2017). Histological Subtypes of Hepatocellular Carcinoma Are Related to Gene Mutations and Molecular Tumour Classification. J. Hepatology 67 (4), 727–738. 10.1016/j.jhep.2017.05.014 28532995

[B13] Capelletti M. M. Manceau H. Puy H. Peoc’h K. (2020). Ferroptosis in Liver Diseases: An Overview. Ijms 21 (14), 4908. 10.3390/ijms21144908 PMC7404091 32664576

[B14] Castro F. Cardoso A. P. Gonçalves R. M. Serre K. Oliveira M. J. (2018). Interferon-Gamma at the Crossroads of Tumor Immune Surveillance or Evasion. Front. Immunol. 9, 847. 10.3389/fimmu.2018.00847 29780381 PMC5945880

[B15] Chen C. P. (2019). Role of Radiotherapy in the Treatment of Hepatocellular Carcinoma. J. Clin. Transl. Hepatol. 7 (2), 1–8. 10.14218/jcth.2018.00060 31293919 PMC6609847

[B16] Chen D. Fan Z. Rauh M. Buchfelder M. Eyupoglu I. Y. Savaskan N. (2017). ATF4 Promotes Angiogenesis and Neuronal Cell Death and Confers Ferroptosis in a xCT-dependent Manner. Oncogene 36 (40), 5593–5608. 10.1038/onc.2017.146 28553953 PMC5633655

[B19] Cullinan S. B. Gordan J. D. Jin J. Harper J. W. Diehl J. A. (2004). The Keap1-BTB Protein Is an Adaptor that Bridges Nrf2 to a Cul3-Based E3 Ligase: Oxidative Stress Sensing by a Cul3-Keap1 Ligase. Mol. Cell Biol. 24 (19), 8477–8486. 10.1128/mcb.24.19.8477-8486.2004 15367669 PMC516753

[B20] Dalton T. P. Dieter M. Z. Yang Y. Shertzer H. G. Nebert D. W. (2000). Knockout of the Mouse Glutamate Cysteine Ligase Catalytic Subunit (Gclc) Gene: Embryonic Lethal when Homozygous, and Proposed Model for Moderate Glutathione Deficiency when Heterozygous. Biochem. Biophysical Res. Commun. 279 (2), 324–329. 10.1006/bbrc.2000.3930 11118286

[B21] Delaney G. Jacob S. Featherstone C. Barton M. (2005). The Role of Radiotherapy in Cancer Treatment: Estimating Optimal Utilization from a Review of Evidence-Based Clinical Guidelines. Cancer 104 (6), 1129–1137. 10.1002/cncr.21324 16080176

[B22] Deng D. X. Chakrabarti S. Waalkes M. P. Cherian M. G. (1998). Metallothionein and Apoptosis in Primary Human Hepatocellular Carcinoma and Metastatic Adenocarcinoma. Histopathology 32 (4), 340–347. 10.1046/j.1365-2559.1998.00348.x 9602331

[B23] Dimaras H. Khetan V. Halliday W. Orlic M. Prigoda N. L. Piovesan B. (2008). Loss of RB1 Induces Non-proliferative Retinoma: Increasing Genomic Instability Correlates with Progression to Retinoblastoma. Hum. Mol. Genet. 17 (10), 1363–1372. 10.1093/hmg/ddn024 18211953

[B24] Dixon S. J. Lemberg K. M. Lamprecht M. R. Skouta R. Zaitsev E. M. Gleason C. E. (2012). Ferroptosis: an Iron-dependent Form of Nonapoptotic Cell Death. Cell 149 (5), 1060–1072. 10.1016/j.cell.2012.03.042 22632970 PMC3367386

[B25] Dixon S. J. Patel D. N. Welsch M. Skouta R. Lee E. D. Hayano M. (2014). Pharmacological Inhibition of Cystine-Glutamate Exchange Induces Endoplasmic Reticulum Stress and Ferroptosis. Elife 3, e02523. 10.7554/eLife.02523 24844246 PMC4054777

[B26] Dolma S. Lessnick S. L. Hahn W. C. Stockwell B. R. (2003). Identification of Genotype-Selective Antitumor Agents Using Synthetic Lethal Chemical Screening in Engineered Human Tumor Cells. Cancer Cell 3 (3), 285–296. 10.1016/s1535-6108(03)00050-3 12676586

[B27] Doorbar J. (2006). Molecular Biology of Human Papillomavirus Infection and Cervical Cancer. Clin. Sci. (Lond) 110 (5), 525–541. 10.1042/cs20050369 16597322

[B28] Dyson N. J. (2016). RB1: a Prototype Tumor Suppressor and an Enigma. Genes Dev. 30 (13), 1492–1502. 10.1101/gad.282145.116 27401552 PMC4949322

[B29] Dzieran J. Fabian J. Feng T. Coulouarn C. Ilkavets I. Kyselova A. (2013). Comparative Analysis of TGF-β/Smad Signaling Dependent Cytostasis in Human Hepatocellular Carcinoma Cell Lines. PLoS One 8 (8), e72252. 10.1371/journal.pone.0072252 23991075 PMC3750029

[B30] Edlich F. (2018). BCL-2 Proteins and Apoptosis: Recent Insights and Unknowns. Biochem. Biophysical Res. Commun. 500 (1), 26–34. 10.1016/j.bbrc.2017.06.190 28676391

[B31] El–Serag H. B. Rudolph K. L. (2007). Hepatocellular Carcinoma: Epidemiology and Molecular Carcinogenesis. Gastroenterology 132 (7), 2557–2576. 10.1053/j.gastro.2007.04.061 17570226

[B32] Fei Z. Lijuan Y. Jing Z. Xi Y. Yuefen P. Shuwen H. (2021). Molecular Characteristics Associated with Ferroptosis in Hepatocellular Carcinoma Progression. Hum. Cell 34 (1), 177–186. 10.1007/s13577-020-00431-w 32936424

[B33] Forcina G. C. Dixon S. J. (2019). GPX4 at the Crossroads of Lipid Homeostasis and Ferroptosis. Proteomics 19 (18), 1800311. 10.1002/pmic.201800311 30888116

[B34] Forman H. J. Zhang H. Rinna A. (2009). Glutathione: Overview of its Protective Roles, Measurement, and Biosynthesis. Mol. Aspects Med. 30 (1-2), 1–12. 10.1016/j.mam.2008.08.006 18796312 PMC2696075

[B35] Franklin C. C. Rosenfeld-Franklin M. E. White C. Kavanagh T. J. Fausto N. (2003). TGFβ1‐induced Suppression of Glutathione Antioxidant Defenses in Hepatocytes: Caspase‐dependent Post-translational and Caspase‐independent Transcriptional Regulatory Mechanisms. FASEB J. 17 (11), 1–23. 10.1096/fj.02-0867fje 12824300

[B36] Gao M. Monian P. Quadri N. Ramasamy R. Jiang X. (2015). Glutaminolysis and Transferrin Regulate Ferroptosis. Mol. Cell 59 (2), 298–308. 10.1016/j.molcel.2015.06.011 26166707 PMC4506736

[B37] Gao M. Yi J. Zhu J. Minikes A. M. Monian P. Thompson C. B. (2019). Role of Mitochondria in Ferroptosis. Mol. Cell 73 (2), 354–363. e353. 10.1016/j.molcel.2018.10.042 30581146 PMC6338496

[B38] Gao R. Kalathur R. K. R. Coto‐Llerena M. Ercan C. Buechel D. Shuang S. (2021). YAP/TAZ and ATF4 Drive Resistance to Sorafenib in Hepatocellular Carcinoma by Preventing Ferroptosis. EMBO Mol. Med. 13 (12), e14351. 10.15252/emmm.202114351 34664408 PMC8649869

[B39] Garrido C. Brunet M. Didelot C. Zermati Y. Schmitt E. Kroemer G. (2006). Heat Shock Proteins 27 and 70: Anti-apoptotic Proteins with Tumorigenic Properties. Cell Cycle 5 (22), 2592–2601. 10.4161/cc.5.22.3448 17106261

[B40] Gaschler M. M. Andia A. A. Liu H. Csuka J. M. Hurlocker B. Vaiana C. A. (2018). FINO(2) Initiates Ferroptosis through GPX4 Inactivation and Iron Oxidation. Nat. Chem. Biol. 14 (5), 507–515. 10.1038/s41589-018-0031-6 29610484 PMC5899674

[B41] Georgopoulos C. Welch W. J. (1993). Role of the Major Heat Shock Proteins as Molecular Chaperones. Annu. Rev. Cell. Biol. 9, 601–634. 10.1146/annurev.cb.09.110193.003125 8280473

[B42] Griffith O. W. Meister A. (1979). Potent and Specific Inhibition of Glutathione Synthesis by Buthionine Sulfoximine (S-N-Butyl Homocysteine Sulfoximine). J. Biol. Chem. 254 (16), 7558–7560. 10.1016/s0021-9258(18)35980-5 38242

[B43] Gumulec J. Raudenska M. Adam V. Kizek R. Masarik M. (2014). Metallothionein - Immunohistochemical Cancer Biomarker: a Meta-Analysis. PLoS One 9 (1), e85346. 10.1371/journal.pone.0085346 24416395 PMC3885711

[B44] Harris I. S. DeNicola G. M. (2020). The Complex Interplay between Antioxidants and ROS in Cancer. Trends Cell Biol. 30 (6), 440–451. 10.1016/j.tcb.2020.03.002 32303435

[B45] Harris I. S. Endress J. E. Coloff J. L. Selfors L. M. McBrayer S. K. Rosenbluth J. M. (2019). Deubiquitinases Maintain Protein Homeostasis and Survival of Cancer Cells upon Glutathione Depletion. Cell Metab. 29 (5), 1166–1181. e1166. 10.1016/j.cmet.2019.01.020 30799286 PMC6506399

[B46] Harris I. S. Treloar A. E. Inoue S. Sasaki M. Gorrini C. Lee K. C. (2015). Glutathione and Thioredoxin Antioxidant Pathways Synergize to Drive Cancer Initiation and Progression. Cancer Cell 27 (2), 211–222. 10.1016/j.ccell.2014.11.019 25620030

[B47] Holmgren A. Lu J. (2010). Thioredoxin and Thioredoxin Reductase: Current Research with Special Reference to Human Disease. Biochem. Biophysical Res. Commun. 396 (1), 120–124. 10.1016/j.bbrc.2010.03.083 20494123

[B48] Imai H. Hirao F. Sakamoto T. Sekine K. Mizukura Y. Saito M. (2003). Early Embryonic Lethality Caused by Targeted Disruption of the Mouse PHGPx Gene. Biochem. Biophysical Res. Commun. 305 (2), 278–286. 10.1016/s0006-291x(03)00734-4 12745070

[B49] Itoh K. Chiba T. Takahashi S. Ishii T. Igarashi K. Katoh Y. (1997). An Nrf2/small Maf Heterodimer Mediates the Induction of Phase II Detoxifying Enzyme Genes through Antioxidant Response Elements. Biochem. Biophysical Res. Commun. 236 (2), 313–322. 10.1006/bbrc.1997.6943 9240432

[B50] Itoh K. Wakabayashi N. Katoh Y. Ishii T. Igarashi K. Engel J. D. (1999). Keap1 Represses Nuclear Activation of Antioxidant Responsive Elements by Nrf2 through Binding to the Amino-Terminal Neh2 Domain. Genes Dev. 13 (1), 76–86. 10.1101/gad.13.1.76 9887101 PMC316370

[B51] Jennis M. Kung C.-P. Basu S. Budina-Kolomets A. Leu J. I.-J. Khaku S. (2016). An African-specific Polymorphism in the TP53 Gene Impairs P53 Tumor Suppressor Function in a Mouse Model. Genes Dev. 30 (8), 918–930. 10.1101/gad.275891.115 27034505 PMC4840298

[B52] Jiang L. Kon N. Li T. Wang S.-J. Su T. Hibshoosh H. (2015). Ferroptosis as a P53-Mediated Activity during Tumour Suppression. Nature 520 (7545), 57–62. 10.1038/nature14344 25799988 PMC4455927

[B53] Junttila M. R. Evan G. I. (2009). p53 - a Jack of All Trades but Master of None. Nat. Rev. Cancer 9 (11), 821–829. 10.1038/nrc2728 19776747

[B54] Kamada T. Tsujii H. Blakely E. A. Debus J. De Neve W. Durante M. (2015). Carbon Ion Radiotherapy in Japan: an Assessment of 20 Years of Clinical Experience. Lancet Oncol. 16 (2), e93–e100. 10.1016/s1470-2045(14)70412-7 25638685

[B55] Kim D. H. Kim W. D. Kim S. K. Moon D. H. Lee S. J. (2020). TGF-β1-mediated Repression of SLC7A11 Drives Vulnerability to GPX4 Inhibition in Hepatocellular Carcinoma Cells. Cell Death Dis. 11 (5), 406. 10.1038/s41419-020-2618-6 32471991 PMC7260246

[B56] Knekt P. Reunanen A. Takkunen H. Aromaa A. Heliövaara M. Hakuunen T. (1994). Body Iron Stores and Risk of Cancer. Int. J. Cancer 56 (3), 379–382. 10.1002/ijc.2910560315 8314326

[B57] Kobayashi A. Kang M.-I. Okawa H. Ohtsuji M. Zenke Y. Chiba T. (2004). Oxidative Stress Sensor Keap1 Functions as an Adaptor for Cul3-Based E3 Ligase to Regulate Proteasomal Degradation of Nrf2. Mol. Cell Biol. 24 (16), 7130–7139. 10.1128/mcb.24.16.7130-7139.2004 15282312 PMC479737

[B58] Kobayashi A. Kang M.-I. Watai Y. Tong K. I. Shibata T. Uchida K. (2006). Oxidative and Electrophilic Stresses Activate Nrf2 through Inhibition of Ubiquitination Activity of Keap1. Mol. Cell Biol. 26 (1), 221–229. 10.1128/mcb.26.1.221-229.2006 16354693 PMC1317630

[B59] Komatsu M. Kurokawa H. Waguri S. Taguchi K. Kobayashi A. Ichimura Y. (2010). The Selective Autophagy Substrate P62 Activates the Stress Responsive Transcription Factor Nrf2 through Inactivation of Keap1. Nat. Cell Biol. 12 (3), 213–223. 10.1038/ncb2021 20173742

[B60] Kong R. Wang N. Han W. Bao W. Lu J. (2021). Ifnγ‐Mediated Repression of System Xc(–) Drives Vulnerability to Induced Ferroptosis in Hepatocellular Carcinoma Cells. J. Leukoc. Biol. 110 (2), 301–314. 10.1002/jlb.3ma1220-815rrr 34318944

[B61] Koppula P. Zhuang L. Gan B. (2021). Cystine Transporter SLC7A11/xCT in Cancer: Ferroptosis, Nutrient Dependency, and Cancer Therapy. Protein Cell 12 (8), 599–620. 10.1007/s13238-020-00789-5 33000412 PMC8310547

[B62] Kwon M.-Y. Park E. Lee S.-J. Chung S. W. (2015). Heme Oxygenase-1 Accelerates Erastin-Induced Ferroptotic Cell Death. Oncotarget 6 (27), 24393–24403. 10.18632/oncotarget.5162 26405158 PMC4695193

[B63] Lang X. Green M. D. Wang W. Yu J. Choi J. E. Jiang L. (2019). Radiotherapy and Immunotherapy Promote Tumoral Lipid Oxidation and Ferroptosis via Synergistic Repression of SLC7A11. Cancer Discov. 9 (12), 1673–1685. 10.1158/2159-8290.cd-19-0338 31554642 PMC6891128

[B64] Laurent-Puig P. Zucman-Rossi J. (2006). Genetics of Hepatocellular Tumors. Oncogene 25 (27), 3778–3786. 10.1038/sj.onc.1209547 16799619

[B65] Lavoie J. N. Hickey E. Weber L. A. Landry J. (1993). Modulation of Actin Microfilament Dynamics and Fluid Phase Pinocytosis by Phosphorylation of Heat Shock Protein 27. J. Biol. Chem. 268 (32), 24210–24214. 10.1016/s0021-9258(20)80512-2 8226968

[B66] Lee M. Jo A. Lee S. Kim J. B. Chang Y. Nam J. Y. (2017). 3-bromopyruvate and Buthionine Sulfoximine Effectively Kill Anoikis-Resistant Hepatocellular Carcinoma Cells. PLoS One 12 (3), e0174271. 10.1371/journal.pone.0174271 28362858 PMC5376082

[B67] Lee Y. Auh S. L. Wang Y. Burnette B. Wang Y. Meng Y. (2009). Therapeutic Effects of Ablative Radiation on Local Tumor Require CD8+ T Cells: Changing Strategies for Cancer Treatment. Blood 114 (3), 589–595. 10.1182/blood-2009-02-206870 19349616 PMC2713472

[B68] Liang C. Zhang X. Yang M. Dong X. (2019). Recent Progress in Ferroptosis Inducers for Cancer Therapy. Adv. Mat. 31 (51), 1904197. 10.1002/adma.201904197 31595562

[B69] Lievens J.-C. Maurice T. (2021). Sigma-1 Receptor: Culprit and Rescuer in Motor Neuron Diseases. Neural Regen. Res. 16 (1), 106–107. 10.4103/1673-5374.286961 32788456 PMC7818861

[B71] Llovet J. M. Ricci S. Mazzaferro V. Hilgard P. Gane E. Blanc J.-F. (2008). Sorafenib in Advanced Hepatocellular Carcinoma. N. Engl. J. Med. 359 (4), 378–390. 10.1056/NEJMoa0708857 18650514

[B72] Long S. Peng F. Song B. Wang L. Chen J. Shang B. (2021). Heat Shock Protein Beta 1 Is a Prognostic Biomarker and Correlated with Immune Infiltrates in Hepatocellular Carcinoma. Ijgm 14, 5483–5492. 10.2147/ijgm.s330608 PMC8439715 34531676

[B73] Louandre C. Ezzoukhry Z. Godin C. Barbare J.-C. Mazière J.-C. Chauffert B. (2013). Iron-dependent Cell Death of Hepatocellular Carcinoma Cells Exposed to Sorafenib. Int. J. Cancer 133 (7), 1732–1742. 10.1002/ijc.28159 23505071

[B74] Louandre C. Marcq I. Bouhlal H. Lachaier E. Godin C. Saidak Z. (2015). The Retinoblastoma (Rb) Protein Regulates Ferroptosis Induced by Sorafenib in Human Hepatocellular Carcinoma Cells. Cancer Lett. 356 (2 Pt B), 971–977. 10.1016/j.canlet.2014.11.014 25444922

[B75] Lu S. C. (2013). Glutathione Synthesis. Biochimica Biophysica Acta (BBA) - General Subj. 1830 (5), 3143–3153. 10.1016/j.bbagen.2012.09.008 PMC3549305 22995213

[B76] Lu S. C. (2009). Regulation of Glutathione Synthesis. Mol. Aspects Med. 30 (1-2), 42–59. 10.1016/j.mam.2008.05.005 18601945 PMC2704241

[B77] Ma Q. (2013). Role of Nrf2 in Oxidative Stress and Toxicity. Annu. Rev. Pharmacol. Toxicol. 53, 401–426. 10.1146/annurev-pharmtox-011112-140320 23294312 PMC4680839

[B78] Marengo A. Rosso C. Bugianesi E. (2016). Liver Cancer: Connections with Obesity, Fatty Liver, and Cirrhosis. Annu. Rev. Med. 67, 103–117. 10.1146/annurev-med-090514-013832 26473416

[B79] Mãrtensson J. Meister A. Mrtensson J. (1991). Glutathione Deficiency Decreases Tissue Ascorbate Levels in Newborn Rats: Ascorbate Spares Glutathione and Protects. Proc. Natl. Acad. Sci. U.S.A. 88 (11), 4656–4660. 10.1073/pnas.88.11.4656 2052548 PMC51724

[B80] Mayhew C. N. Carter S. L. Fox S. R. Sexton C. R. Reed C. A. Srinivasan S. V. (2007). RB Loss Abrogates Cell Cycle Control and Genome Integrity to Promote Liver Tumorigenesis. Gastroenterology 133 (3), 976–984. 10.1053/j.gastro.2007.06.025 17854601

[B81] Meister A. (1995). Mitochondrial Changes Associated with Glutathione Deficiency. Biochimica Biophysica Acta (BBA) - Mol. Basis Dis. 1271 (1), 35–42. 10.1016/0925-4439(95)00007-q 7599223

[B82] Meuwissen R. Linn S. C. Linnoila R. I. Zevenhoven J. Mooi W. J. Berns A. (2003). Induction of Small Cell Lung Cancer by Somatic Inactivation of Both Trp53 and Rb1 in a Conditional Mouse Model. Cancer Cell 4 (3), 181–189. 10.1016/s1535-6108(03)00220-4 14522252

[B84] Motaghed M. Al-Hassan F. M. Hamid S. S. (2014). Thymoquinone Regulates Gene Expression Levels in the Estrogen Metabolic and Interferon Pathways in MCF7 Breast Cancer Cells. Int. J. Mol. Med. 33 (1), 8–16. 10.3892/ijmm.2013.1563 24270600 PMC3868490

[B85] Munakata T. Liang Y. Kim S. McGivern D. R. Huibregtse J. Nomoto A. (2007). Hepatitis C Virus Induces E6AP-dependent Degradation of the Retinoblastoma Protein. PLoS Pathog. 3 (9), e139–1347. 10.1371/journal.ppat.0030139 PMC2323300 17907805

[B86] Nagelkerke A. Bussink J. van der Kogel A. J. Sweep F. C. G. J. Span P. N. (2013). The PERK/ATF4/LAMP3-arm of the Unfolded Protein Response Affects Radioresistance by Interfering with the DNA Damage Response. Radiotherapy Oncol. 108 (3), 415–421. 10.1016/j.radonc.2013.06.037 23891100

[B87] Nguyen T. Sherratt P. J. Pickett C. B. (2003). Regulatory Mechanisms Controlling Gene Expression Mediated by the Antioxidant Response Element. Annu. Rev. Pharmacol. Toxicol. 43, 233–260. 10.1146/annurev.pharmtox.43.100901.140229 12359864

[B88] Nickoloff J. A. (2015). Photon, Light Ion, and Heavy Ion Cancer Radiotherapy: Paths from Physics and Biology to Clinical Practice. Ann. Transl. Med. 3 (21), 336. 10.3978/j.issn.2305-5839.2015.12.18 26734646 PMC4691008

[B89] Okuno M. Adachi S. Kozawa O. Shimizu M. Yasuda I. (2016). The Clinical Significance of Phosphorylated Heat Shock Protein 27 (HSPB1) in Pancreatic Cancer. Ijms 17 (1), 137. 10.3390/ijms17010137 PMC4730376 26805817

[B90] Pal A. Fontanilla D. Gopalakrishnan A. Chae Y.-K. Markley J. L. Ruoho A. E. (2012). The Sigma-1 Receptor Protects against Cellular Oxidative Stress and Activates Antioxidant Response Elements. Eur. J. Pharmacol. 682 (1-3), 12–20. 10.1016/j.ejphar.2012.01.030 22381068 PMC3314091

[B91] Piccolo S. Dupont S. Cordenonsi M. (2014). The Biology of YAP/TAZ: Hippo Signaling and beyond. Physiol. Rev. 94 (4), 1287–1312. 10.1152/physrev.00005.2014 25287865

[B92] Poss K. D. Tonegawa S. (1997). Heme Oxygenase 1 Is Required for Mammalian Iron Reutilization. Proc. Natl. Acad. Sci. U.S.A. 94 (20), 10919–10924. 10.1073/pnas.94.20.10919 9380735 PMC23531

[B93] Prigge J. R. Coppo L. Martin S. S. Ogata F. Miller C. G. Bruschwein M. D. (2017). Hepatocyte Hyperproliferation upon Liver-specific Co-disruption of Thioredoxin-1, Thioredoxin Reductase-1, and Glutathione Reductase. Cell Rep. 19 (13), 2771–2781. 10.1016/j.celrep.2017.06.019 28658624 PMC5730093

[B94] Recalcati S. Minotti G. Cairo G. (2010). Iron Regulatory Proteins: from Molecular Mechanisms to Drug Development. Antioxidants Redox Signal. 13 (10), 1593–1616. 10.1089/ars.2009.2983 20214491

[B95] Rogers L. K. Tamura T. Rogers B. J. Welty S. E. Hansen T. N. Smith C. V. (2004). Analyses of Glutathione Reductase Hypomorphic Mice Indicate a Genetic Knockout. Toxicol. Sci. 82 (2), 367–373. 10.1093/toxsci/kfh268 15342956

[B96] Sasaki H. Sato H. Kuriyama-Matsumura K. Sato K. Maebara K. Wang H. (2002). Electrophile Response Element-Mediated Induction of the Cystine/glutamate Exchange Transporter Gene Expression. J. Biol. Chem. 277 (47), 44765–44771. 10.1074/jbc.M208704200 12235164

[B98] Shimada K. Skouta R. Kaplan A. Yang W. S. Hayano M. Dixon S. J. (2016). Global Survey of Cell Death Mechanisms Reveals Metabolic Regulation of Ferroptosis. Nat. Chem. Biol. 12 (7), 497–503. 10.1038/nchembio.2079 27159577 PMC4920070

[B99] Si M. Lang J. (2018). The Roles of Metallothioneins in Carcinogenesis. J. Hematol. Oncol. 11 (1), 107. 10.1186/s13045-018-0645-x 30139373 PMC6108115

[B100] Siegel A. B. Olsen S. K. Magun A. Brown R. S. Jr. (2010). Sorafenib: where Do We Go from Here? Hepatology 52 (1), 360–369. 10.1002/hep.23633 20578152 PMC3605708

[B101] Singal A. G. El-Serag H. B. (2015). Hepatocellular Carcinoma from Epidemiology to Prevention: Translating Knowledge into Practice. Clin. Gastroenterology Hepatology 13 (12), 2140–2151. 10.1016/j.cgh.2015.08.014 PMC4618036 26284591

[B102] Stevens R. G. Graubard B. I. Micozzi M. S. Neriishi K. Blumberg B. S. (1994). Moderate Elevation of Body Iron Level and Increased Risk of Cancer Occurrence and Death. Int. J. Cancer 56 (3), 364–369. 10.1002/ijc.2910560312 8314323

[B103] Sun T. Chi J.-T. (2021). Regulation of Ferroptosis in Cancer Cells by YAP/TAZ and Hippo Pathways: The Therapeutic Implications. Genes & Dis. 8 (3), 241–249. 10.1016/j.gendis.2020.05.004 PMC8093643 33997171

[B104] Sun X. Niu X. Chen R. He W. Chen D. Kang R. (2016a). Metallothionein‐1G Facilitates Sorafenib Resistance through Inhibition of Ferroptosis. Hepatology 64 (2), 488–500. 10.1002/hep.28574 27015352 PMC4956496

[B105] Sun X. Ou Z. Chen R. Niu X. Chen D. Kang R. (2016b). Activation of the P62-Keap1-NRF2 Pathway Protects against Ferroptosis in Hepatocellular Carcinoma Cells. Hepatology 63 (1), 173–184. 10.1002/hep.28251 26403645 PMC4688087

[B106] Sun X. Ou Z. Xie M. Kang R. Fan Y. Niu X. (2015). HSPB1 as a Novel Regulator of Ferroptotic Cancer Cell Death. Oncogene 34 (45), 5617–5625. 10.1038/onc.2015.32 25728673 PMC4640181

[B107] Sung H. Ferlay J. Siegel R. L. Laversanne M. Soerjomataram I. Jemal A. (2021). Global Cancer Statistics 2020: GLOBOCAN Estimates of Incidence and Mortality Worldwide for 36 Cancers in 185 Countries. CA A Cancer J. Clin. 71 (3), 209–249. 10.3322/caac.21660 33538338

[B108] Tang B. Zhu J. Li J. Fan K. Gao Y. Cheng S. (2020). The Ferroptosis and Iron-Metabolism Signature Robustly Predicts Clinical Diagnosis, Prognosis and Immune Microenvironment for Hepatocellular Carcinoma. Cell Commun. Signal 18 (1), 174. 10.1186/s12964-020-00663-1 33115468 PMC7592541

[B109] Tenhunen R. Marver H. S. Schmid R. (1969). Microsomal Heme Oxygenase. Characterization of the Enzyme. J. Biol. Chem. 244 (23), 6388–6394. 10.1016/s0021-9258(18)63477-5 4390967

[B110] Thariat J. Hannoun-Levi J.-M. Sun Myint A. Vuong T. Gérard J.-P. (2013). Past, Present, and Future of Radiotherapy for the Benefit of Patients. Nat. Rev. Clin. Oncol. 10 (1), 52–60. 10.1038/nrclinonc.2012.203 23183635

[B111] Tsurusaki S. Tsuchiya Y. Koumura T. Nakasone M. Sakamoto T. Matsuoka M. (2019). Hepatic Ferroptosis Plays an Important Role as the Trigger for Initiating Inflammation in Nonalcoholic Steatohepatitis. Cell Death Dis. 10 (6), 449. 10.1038/s41419-019-1678-y 31209199 PMC6579767

[B113] Wakabayashi N. Dinkova-Kostova A. T. Holtzclaw W. D. Kang M.-I. Kobayashi A. Yamamoto M. (2004). Protection against Electrophile and Oxidant Stress by Induction of the Phase 2 Response: Fate of Cysteines of the Keap1 Sensor Modified by Inducers. Proc. Natl. Acad. Sci. U.S.A. 101 (7), 2040–2045. 10.1073/pnas.0307301101 14764894 PMC357048

[B114] Wang J. Shanmugam A. Markand S. Zorrilla E. Ganapathy V. Smith S. B. (2015). Sigma 1 Receptor Regulates the Oxidative Stress Response in Primary Retinal Müller Glial Cells via NRF2 Signaling and System Xc(−), the Na(+)-independent Glutamate-Cystine Exchanger. Free Radic. Biol. Med. 86, 25–36. 10.1016/j.freeradbiomed.2015.04.009 25920363 PMC4554890

[B115] Wang Q. Bin C. Xue Q. Gao Q. Huang A. Wang K. (2021). GSTZ1 Sensitizes Hepatocellular Carcinoma Cells to Sorafenib-Induced Ferroptosis via Inhibition of NRF2/GPX4 axis. Cell Death Dis. 12 (5), 426. 10.1038/s41419-021-03718-4 33931597 PMC8087704

[B117] Wang Z. Li Z. Ye Y. Xie L. Li W. (2016). Oxidative Stress and Liver Cancer: Etiology and Therapeutic Targets. Oxidative Med. Cell. Longev. 2016, 1–10. 10.1155/2016/7891574 PMC5121466 27957239

[B118] Westbrook A. M. Wei B. Hacke K. Xia M. Braun J. Schiestl R. H. (2012). The Role of Tumour Necrosis Factor- and Tumour Necrosis Factor Receptor Signalling in Inflammation-Associated Systemic Genotoxicity. Mutagenesis 27 (1), 77–86. 10.1093/mutage/ger063 21980144 PMC3241942

[B119] Wu J. Liu T. Rios Z. Mei Q. Lin X. Cao S. (2017). Heat Shock Proteins and Cancer. Trends Pharmacol. Sci. 38 (3), 226–256. 10.1016/j.tips.2016.11.009 28012700

[B120] Yagoda N. von Rechenberg M. Zaganjor E. Bauer A. J. Yang W. S. Fridman D. J. (2007). RAS-RAF-MEK-dependent Oxidative Cell Death Involving Voltage-dependent Anion Channels. Nature 447 (7146), 865–869. 10.1038/nature05859 PMC3047570 17568748

[B121] Yamada N. Karasawa T. Kimura H. Watanabe S. Komada T. Kamata R. (2020). Ferroptosis Driven by Radical Oxidation of N-6 Polyunsaturated Fatty Acids Mediates Acetaminophen-Induced Acute Liver Failure. Cell Death Dis. 11 (2), 144. 10.1038/s41419-020-2334-2 32094346 PMC7039960

[B122] Yang W. S. Kim K. J. Gaschler M. M. Patel M. Shchepinov M. S. Stockwell B. R. (2016). Peroxidation of Polyunsaturated Fatty Acids by Lipoxygenases Drives Ferroptosis. Proc. Natl. Acad. Sci. U.S.A. 113 (34), E4966–E4975. 10.1073/pnas.1603244113 27506793 PMC5003261

[B123] Yang W. S. SriRamaratnam R. Welsch M. E. Shimada K. Skouta R. Viswanathan V. S. (2014). Regulation of Ferroptotic Cancer Cell Death by GPX4. Cell 156 (1-2), 317–331. 10.1016/j.cell.2013.12.010 24439385 PMC4076414

[B124] Yang W. S. Stockwell B. R. (2008). Synthetic Lethal Screening Identifies Compounds Activating Iron-dependent, Nonapoptotic Cell Death in Oncogenic-RAS-Harboring Cancer Cells. Chem. Biol. 15 (3), 234–245. 10.1016/j.chembiol.2008.02.010 18355723 PMC2683762

[B125] Zanconato F. Cordenonsi M. Piccolo S. (2016). YAP/TAZ at the Roots of Cancer. Cancer Cell 29 (6), 783–803. 10.1016/j.ccell.2016.05.005 27300434 PMC6186419

[B126] Zhang Y. Tao X. Jin G. Jin H. Wang N. Hu F. (2016). A Targetable Molecular Chaperone Hsp27 Confers Aggressiveness in Hepatocellular Carcinoma. Theranostics 6 (4), 558–570. 10.7150/thno.14693 26941848 PMC4775865

[B127] Zhang Z. Yao Z. Wang L. Ding H. Shao J. Chen A. (2018). Activation of Ferritinophagy Is Required for the RNA-Binding Protein ELAVL1/HuR to Regulate Ferroptosis in Hepatic Stellate Cells. Autophagy 14 (12), 2083–2103. 10.1080/15548627.2018.1503146 30081711 PMC6984765

[B128] Zheng X. Liu B. Liu X. Li P. Zhang P. Ye F. (2022). PERK Regulates the Sensitivity of Hepatocellular Carcinoma Cells to High-LET Carbon Ions via Either Apoptosis or Ferroptosis. J. Cancer 13 (2), 669–680. 10.7150/jca.61622 35069910 PMC8771512

